# Screening for colorectal cancer with FOBT, virtual colonoscopy and optical colonoscopy: study protocol for a randomized controlled trial in the Florence district (SAVE study)

**DOI:** 10.1186/1745-6215-14-74

**Published:** 2013-03-15

**Authors:** Lapo Sali, Grazia Grazzini, Francesca Carozzi, Guido Castiglione, Massimo Falchini, Beatrice Mallardi, Paola Mantellini, Leonardo Ventura, Daniele Regge, Marco Zappa, Mario Mascalchi, Stefano Milani

**Affiliations:** 1 Radiodiagnostic Section, Department of Clinical Physiopathology, University of Florence, Viale G. Pieraccini 6, 50139 Florence, Italy; 2 Cancer Research and Prevention Institute (ISPO), Via Cosimo il Vecchio 2, 50139 Florence, Italy; 3 Radiology Unit, Institute for Cancer Research and Treatment, Strada Provinciale 142, Km 3,95, Candiolo, Turin, 10060, Italy; 4 Gastroenterology Section, Department of Clinical Physiopathology, University of Florence, Viale G. Pieraccini 6, 50139 Florence, Italy

**Keywords:** Colorectal cancer, Screening, FOBT, Colonoscopy, CT colonography, Virtual colonoscopy, CAD, Biological bank

## Abstract

**Background:**

Colorectal cancer (CRC) is the most frequent cancer in Europe. Randomized clinical trials demonstrated that screening with fecal occult blood test (FOBT) reduces mortality from CRC. Accordingly, the European Community currently recommends population-based screening with FOBT. Other screening tests, such as computed tomography colonography (CTC) and optical colonoscopy (OC), are highly accurate for examining the entire colon for adenomas and CRC. Acceptability represents a critical determinant of the impact of a screening program. We designed a randomized controlled trial to compare participation rate and diagnostic yield of FOBT, CTC with computer-aided diagnosis, and OC as primary tests for population-based screening.

**Methods/Design:**

A total of 14,000 subjects aged 55 to 64 years, living in the Florence district and never screened for CRC, will be randomized in three arms: group 1 (5,000 persons) invited to undergo CTC (divided into: subgroup 1A with reduced cathartic preparation and subgroup 1B with standard bowel preparation); group 2 (8,000 persons) invited to undergo a biannual FOBT for three rounds; and group 3 (1,000 persons) invited to undergo OC. Subjects of each group will be invited by mail to undergo the selected test. All subjects with a positive FOBT or CTC test (that is, mass or at least one polyp ≥6 mm) will be invited to undergo a second-level OC. Primary objectives of the study are to compare the participation rate to FOBT, CTC and OC; to compare the detection rate for cancer or advanced adenomas of CTC versus three rounds of biannual FOBT; to evaluate referral rate for OC induced by primary CTC versus three rounds of FOBT; and to estimate costs of the three screening strategies. A secondary objective of the study is to create a biological bank of blood and stool specimens from subjects undergoing CTC and OC.

**Discussion:**

This study will provide information about participation/acceptability, diagnostic yield and costs of screening with CTC in comparison with the recommended test (FOBT) and OC.

**Trial registration:**

ClinicalTrials.gov Identifier: NCT01651624.

## Background

Colorectal cancer (CRC) is the most frequent cancer in Europe. In 2008, 436,000 persons were diagnosed with CRC and 212,000 persons died from the disease [1]. In Italy, between 2003 and 2005, CRC was the fourth most frequent neoplastic disease in males and the third in females. It was also the second leading cause of cancer death both in males and females. In Italy 20,457 new cases of CRC in males and 17,276 in females are estimated to occur every year [2].

A combination of early detection and improvement of treatment increases survival rate for CRC [3]. In particular, the removal of its precursor lesion (adenoma) decreases both the incidence and mortality of CRC [4], and population screening of asymptomatic average risk persons reduces the incidence and mortality rate for CRC [5-10].

Currently, several potential screening tests including fecal occult blood test (FOBT), flexible sigmoidoscopy (FS), optical colonoscopy (OC) and computed tomography colonography (CTC) are available and variably recommended by scientific societies [11,12]. FOBT is a simple, cheap and safe laboratory test that relies on the assumption that asymptomatic CRC and large adenomas may bleed. For this reason it is affected by false negative results due to, for example, incorrect storage of sample or drug assumption, and is affected by false positive results due to hemorrhoids or diet and medications in case of guaiac test [11]. All the other examinations, varying in complexity and costs, allow direct depiction of CRC or adenomas but suffer procedural risks (that is, complications, such as colonic perforation) or biological risks (that is, ionizing radiation exposure) [11].

So far, three randomized clinical trials (RCT) demonstrated that screening with FOBT reduces mortality from CRC in comparison to a no-screening condition [5-7]. Moreover fecal immunochemical test (FIT) showed a better sensitivity-specificity ratio and cost-effectiveness than guaiac test for search of fecal occult blood [13-15].

Screening programs with immunochemical FOBT have been implemented in several regions of Italy [16] and in the Tuscany region such a program has been active since 2000 under the coordination of the Cancer Research and Prevention Institute (ISPO) [17]. ISPO is an institution of the Tuscany Health Service, which aims to organize, realize and monitor clinical and research activities in population-based screening programs for breast, cervical and colorectal cancer in the Tuscany region of Italy [18].

CRC screening is offered to all subjects aged 50 to 70 years who are invited by mail every second year to undergo immunochemical FOBT. Subjects with a negative FOBT are notified of their test results by mail and advised to repeat screening after two years. Subjects with a positive test are invited to undergo total OC [17].

Unlike FS, both OC and CTC enable exploration of the entire colon length. OC is widely accepted as the gold standard procedure for detection of colorectal neoplasia, but there is no evidence that OC screening is effective in reducing mortality for CRC, although indirect data show that this strategy may contribute to a 76 to 90% decrease of the incidence for CRC [4]. Moreover, screening with OC in selected cohorts of subjects by detection and removal of most advanced adenomas could allow long screening intervals [19,20]. The major disadvantages of OC as a screening test are its complications, including bleeding and perforation, and the discomfort due to both full bowel preparation and the procedure itself [21]. As a matter of fact, attendance at an OC as primary screening test for CRC is quite low [22].

CTC is a minimally invasive examination. A recent meta-analysis showed that CTC has 96.1% sensitivity for CRC [23]. In two large trials conducted on asymptomatic individuals at average risk for CRC, CTC showed high per-patient accuracy for adenomas larger than 10 mm with 92.2% to 96% sensitivity and 86% to 96% specificity [24,25]. In another large study the diagnostic yield for advanced neoplasia of CTC (3.2%) was similar to that of OC (3.4%) [26]. Notably, the risk of complications from CTC is extremely low, particularly in asymptomatic subjects [27-29].

The need of bowel preparation for CTC can adversely influence acceptability of this screening test. However limited bowel preparations reducing the subject’s discomfort are now available [30]. A distinctive disadvantage of CTC is exposure of the subject to ionizing radiation, but use of low-dose protocols limits this concern [31].

A distinguishing feature of CTC as compared to FS and OC is that it enables visualization of abdominal organs external to the colon. The prevalence of significant extracolonic findings can be quite high and was about 6% in a large screening cohort [32]. Admittedly, detection of extracolonic findings can be beneficial but can also generate anxiety in the subject and induce further costly diagnostic examinations.

A final operational aspect that has to be considered when CTC is used as primary screening test for CRC is that CTC reading may be time consuming and fatiguing for the radiologist. Computer-aided diagnosis (CAD) systems have been developed in order to help the radiologist’s detection of colorectal polypoid lesions. The use of CAD as a second reader improves sensitivity of CTC [33]. The use of CAD as a first reader could be particularly useful in a screening setting, and available data suggest that CAD as first reader is associated with similar per-polyp and per-patient sensitivity as compared to unassisted reading [34].

The optimal screening test for CRC has not been established yet. FS and FOBT reduce incidence and/or mortality for CRC. However, they do not explore the entire colon and their ability to detect adenomas is not completely satisfactory.

To date, two RCTs investigated participation rate in CTC population screening [35,36]. In the Australian trial, participation rate was 28.4% [35]. In the Dutch study 8,844 subjects aged 50 to 75 years were randomly allocated (2:1) to be invited for primary screening for CRC by OC or CTC. Participation rate in the CTC group was significantly higher than participation in the OC group (34% versus 22%), whereas diagnostic yield for advanced neoplasia was similar for the two screening tests [36].

We designed and report herein the protocol of a RCT comparing participation rate, diagnostic yield and costs of a population-based screening program for colorectal cancer with FOBT, CTC and OC in the Florence district of Italy (SAVE study).

## Methods/Design

### Objectives

The primary objectives of this study are:

1. To compare the participation rate to FOBT, CTC and OC as primary screening tests in a population-based program for CRC.

2. To compare the participation rate to CTC with reduced cathartic preparation versus CTC with standard bowel preparation.

3. To compare the detection rate for cancer or advanced adenomas of CTC with CAD versus three rounds of FOBT every second year.

4. To evaluate the referral rate for OC induced by primary CTC versus three rounds of FOBT every second year.

5. To compare the costs of the three screening strategies using an activity-based costing model.

The secondary objectives of this study are:

1. To compare the expected and perceived discomfort of CTC and OC as assessed with a structured questionnaire.

2. To evaluate the rate of complications in each screened group.

3. To create a biological bank of blood and stool specimens from subjects who undergo CTC, screening OC and second-level OC.

### Study design and population

**Figure 1:**
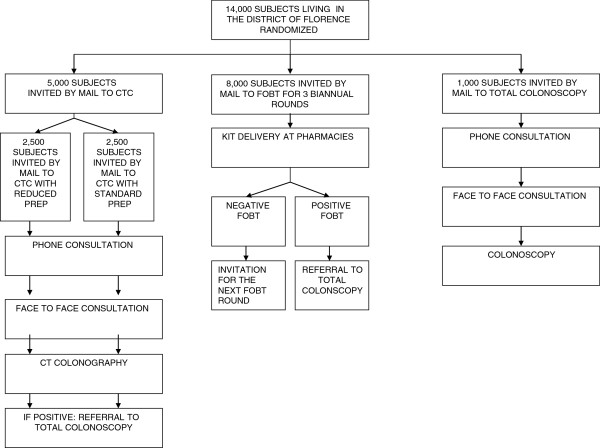
**Study design.** Flow chart.

The study will be a prospective RCT with three arms (Figure 1). The target of our study will be a cohort of 14,000 subjects aged 55 to 64 years who live in the Florence district of Italy and have never been screened before, who will be drawn from the population files of the Florence Municipality, and who will be randomized into three groups of different sizes as follows:

1. Group 1 will consist of 5,000 persons invited to undergo CTC. This group will be divided into two subgroups; subgroup 1A will be invited to undergo CTC with reduced cathartic preparation (see below) and subgroup 1B that will be invited to undergo CTC with standard bowel preparation (see below).

2. Group 2 will consist of 8,000 persons invited to undergo FOBT every second year for three rounds, according to the current procedure of the regional screening protocol.

3. Group 3 will consist of 1,000 persons invited to undergo OC.

Simple randomization will be performed by personnel of the epidemiology unit of ISPO in a single procedure using STATA software version 12 (http://www.stata.com). Randomization of married people will be forced; that is, individuals will be assigned to the same arm of their husband/wife, to prevent subjects’ requests to be allocated to another group. Invitees and investigators will not be masked from the allocation.

### Invitation procedure

A specialized database will be created at ISPO and will be used for the invitation procedure, which will start in September 2012 and will end in December 2013. All target subjects will be invited to the selected screening test by mail.

Individuals of each group will receive an invitation letter and an information leaflet. Four different information leaflets will be used, one for each group/subgroup. The leaflets contain information about CRC, importance of screening, and advantages and possible risks of the selected test. The leaflets are based on those used in the regional screening program and underwent an internal validation procedure using the ‘focus group technique’, which involved ten ISPO employees aged 55 to 64 years. The invitation letter for the FOBT group also contains instructions on how to collect the test kit at the nearest pharmacy. The invitation letter for the CTC and the OC also contains a phone number and an email address for the screening center. All invitees have the option to call or to send an email to the screening center in order to make an appointment for a consultation. All non-responders will receive a reminder by mail after three months. Non-responders to the reminder will be invited to undergo FOBT according to current screening procedure.

### Consultation

Subjects who accept an invitation for CTC or OC will have a preliminary consultation at the screening center with a trained nurse. During the consultation subjects will be informed about the study protocol, the screening examination, the bowel preparation, and management in case of positive results. Moreover, information concerning biological banking will be given. Informed consent for participation to the study and for biological banking will be requested at the end of the consultation. Subjects will then be scheduled for the selected examination (CTC or OC).

### Exclusion criteria

Before randomization, the selected cohort of subjects will be matched with the archive of ISPO in order to exclude from the study subjects with a personal history of CRC or with advanced adenomas, inflammatory bowel disease, complete OC in the last five years, or FOBT in the last two years. Also, subjects who report one of the above-mentioned conditions at the preliminary consultation would be excluded from the study.

### Primary screening tests

#### Fecal occult blood test

The FOBT screening test adopted is OC-SENSOR DIANA (Eiken Chemical Co., Tokyo, Japan), a quantitative, completely automated immunochemical test, based on latex agglutination. Positivity threshold is set at 100 ng hemoglobin/ml of sample solution. According to the current screening procedure, subjects are invited to collect the test kit in any of the pharmacies of the district of Florence and to return fecal specimens to one of the seven collection points in town. Specimens are processed at the ISPO laboratory, usually within one week.

#### Computed tomography colonography

Subjects of subgroup 1A will receive a reduced cathartic preparation consisting of a three-day, low fiber diet and the administration of 13.8 g of macrogol 3,350 (MOVICOL, Norgine, Milano, Italy) diluted in a glass of water at the three main meals for three days before the examination. Subjects of subgroup 1B will undergo a standard bowel preparation consisting of a five-day, low fiber diet and a two-liter solution of polyethylene glycol (MOVIPREP, Norgine, Milano, Italy) followed by two liters of clear liquid the day before the examination.

In both subgroups fecal tagging will be obtained with 70 ml of iodinated oral contrast agent (GASTROGRAFIN, Bayer Schering Pharma AG, Berlin, Germany) administered three hours before the procedure. Colonic distension will be performed by a radiologist using an automatic carbon-dioxide insufflator (PROTOCO2L, Bracco, EZEM, Lake Success, USA), after intravenous administration of 20 mg of scopolamine butylbromide (BUSCOPAN, Boehringer Ingelheim Italia, Milan, Italy), if not contraindicated (for example, hypersensitivity to scopolamine butylbromide or to any of the product inactive ingredients; untreated narrow angle glaucoma; or prostatic hypertrophy with urinary retention).

CTC will be performed in two different hospitals in the Florence district (Azienda Ospedaliero Universitaria Careggi, Nuovo Ospedale S. Giovanni di Dio). Then CTC data will be transferred to a centralized reading center for interpretation, through the RIS/PACS metropolitan area network using uncompressed Digital Imaging and Communications in Medicine (**DICOM**) standard format.

CTC will be performed with 16 or 64 rows of detectors CT scanner using a low-dose protocol (16 rows of detectors: collimation 16 × 0.75 mm, section width 1 mm, pitch 1.25, rotation time 0.5 s, 120 kVp, 50 effective mAs; 64 rows of detectors: collimation 32 × 0.6 mm, section width 1 mm, pitch 1.4, rotation time 0.5 s, 120 kVp, 50 effective mAs). The radiologist and technician performing the CTC acquisition will receive special training for the colonic distension and scanning procedure.

In the reading center each CTC will be evaluated by a radiologist using CAD as the first reader (CADCOLON, Im3D, Turin, Italy). First, the radiologist will examine the polyp candidates proposed by CAD using 2D images with 3D view for problem solving. Then he/she will perform a quick unassisted 2D reading, again supplemented by 3D for problem solving, to look for lesions missed by CAD. Each radiologist involved in the CTC evaluation is required both to have read at least 300 examinations and to have completed a qualified test based on a series of 30 endoscopically verified CTCs achieving per-patient sensitivity and specificity of at least 90% for lesions ≥6 mm. Two to five CTC readers will be recruited for the study.

CTC-detected lesions will be recorded according to their morphology (sessile, pedunculated, flat, vegetating, stenosing), location (rectum, sigmoid, descending, transverse, ascending and cecum) and maximum diameter on 2D images. Flat lesions will be defined as a polyp with height less than 3 mm above the mucosal surface [37]. Polyps smaller than 6 mm will be annotated but will not be mentioned in the final report. CTCs will be reported according to the CT Colonography Reporting and Data System (C-RADS) classification [37]. No special effort will be performed to look for extracolonic findings. Relevant extracolonic findings (C-RADS E3, E4) seen by the radiologist during quick axial images scrolling will be annotated and communicated to the subject in the screening center [37]. Less important extracolonic findings (for example, gallstones) will not be reported. Diagnostic examinations generated by relevant extracolonic findings will not be recorded. CTC reading times will be annotated.

#### Optical colonoscopy

All primary screening colonoscopies will be performed at the endoscopy unit of the ISPO by the same endoscopist with particular experience in screening. All participants will be prepared by low fiber diet for five days and by oral intake of two liter of a polyethylene glycol solution (MOVIPREP, Norgine, Milan, Italy) followed by two liter of clear liquid the day before the examination. OC will be performed under deep sedation with Propofol (DIPRIVAN, AstraZeneca, Milan, Italy), unless deep sedation is refused by the subject. Sedation will be administered by an anesthesiologist who will always be present in the endoscopy room during examinations. All detected lesions will be measured with open biopsy forceps and annotated according to size, macroscopic aspect (sessile, pedunculated, flat, vegetating, stenosing, or ulcerated), and localization (rectum, sigmoid, descending, transverse, ascending and cecum). Definition of flat lesions will be the same as used for CTC.

#### Pathology

All colorectal lesions will be defined according to the WHO criteria [38] as follows: hyperplastic, serrated, tubular, tubular-villous or carcinoma lesion. Advanced adenoma is defined as any adenoma greater than 9 mm and/or with a villous histological component greater than 20%, and/or with severe dysplasia. One or two experienced gastrointestinal pathologists will evaluate histopathology.

#### Complications

Complications of CTC and OC that occur within 30 days after the procedures will be recorded. All complications will be detailed according to type, timing, severity, treatment and outcome. Complications that arise during CTC and OC, or immediately afterward, will be annotated in the study database. Information about subsequent complications will be collected through self-reporting, telephonic questionnaire administered one month after the examination and hospital discharge database.

#### Follow up

##### Fecal occult blood test

All subjects with a negative FOBT will receive a mail notification of the result with the indication to repeat the FOBT after two years. All subjects with a positive FOBT will be invited to undergo OC.

##### Computed tomography colonography

Participants with no lesions or with polyps <6 mm at CTC will be classified as negative. They will be informed of the result by mail within three weeks. This cohort of subjects will be scheduled for invitation to undergo a FOBT after five years. In the case of CTC revealing at least one polyp of 6 mm (C-RADS 2), or larger lesions (C-RADS 3 to 4), subjects will be contacted by phone within two weeks and invited to undergo OC. Subjects with relevant extracolonic findings will be contacted by phone and invited to the screening center for a consultation with the radiologist.

##### Optical colonoscopy

Participants undergoing OC will be informed about the result immediately after the procedure. In case of polyps or cancer, histopathology assessment will provide the definitive diagnosis, and participants will be informed about the results in three weeks. In case of cancer, subjects will be referred for treatment and follow-up.

### Second level colonoscopy

OC in subjects with a positive FOBT or CTC will be performed at the endoscopy unit of the ISPO by the same operator and according to the procedure detailed above. All individuals will be informed about the result of the examination using the same procedure as primary screening OC. Subjects with a positive CTC or FOBT who refuse OC assessment will receive a reminder letter containing information about the need for further diagnostic examination. In these cases the option of follow-up CTC will not be offered.

### Biological banking

A secondary objective of the study is creation of a biological bank, which will be undertaken as indicated below:

1. In the CTC group, blood samples will be collected in all subjects who consent to it. Moreover, stool specimens will be collected from subjects with a positive CTC referred to OC.

2. In the FOBT group, all positive subjects referred to OC will be asked to undergo blood and stool sampling at the time of the latter.

3. In the OC group, all attendees will be requested to undergo blood and stool sampling.

Pathological specimens of subjects surgically treated will be collected. Informed consent for sampling and banking will be obtained, according to the European Community directives [39]. All samples will be immediately transferred and stored at −80°C in dedicated freezers at the ISPO. A database will be created in order to manage the stored samples and related clinical information.

### Questionnaires

Acceptability of the test represents a critical determinant of the expected screening benefit [40]. Therefore, we will investigate several issues related to the proposed screening test through structured questionnaires administered at three time points to all participants of the CTC group and of the OC group. First, a questionnaire evaluating discomfort due to bowel preparation and subject’s expectations for the selected examination will be administered immediately before CTC and primary screening OC. A second questionnaire investigating procedural discomfort will be administered immediately after CTC and OC. A third questionnaire investigating all aspects of the screening procedure will be administered by telephonic interview one month after the examination.

### Ethical approval

The study protocol was approved by the Ethical Committee of the Local Health Unit of Florence (Azienda Sanitaria Firenze), number 432/2010/OSS/C.E.L.

### Data analyses

Statistical analyses will be performed using the STATA software version 12.0 and will be based both on the intention-to-treat and the per-protocol principle. Notably, in the intention-to-treat analysis, subjects with a positive CTC who refuse to undergo colonoscopy will be classified as CTC pathway ‘negative’ for advanced adenomas.

We will calculate the participation rate for each screening procedure as the number of participants undergoing the screening test relative to the total number of invitees. Using the chi-square test we will assess if the CTC group will achieve a statistically significant difference in participation as compared to the OC group. Additionally, we will test to determine if better participation will be achieved with reduced as compared to standard cathartic bowel preparation for CTC.

The detection rate of the screening test is defined as the proportion of subjects with screen detected cancer and/or advanced adenoma over the total number of subjects screened. The most advanced detected lesion per screened subject will be used to calculate the detection rate. We will test to see if a statistically significant difference exists between the detection rate of a single round of CTC and the cumulative detection rate of three rounds of FOBT.

Finally we will compute the referral rate for CTC defined as the proportion of subjects attending screening CTC who, being positive to CTC, will be invited to undergo OC.

### Sample size

#### Comparison of CTC versus OC with endpoint ‘compliance to the invitation’

A total of 1,000 people were invited to participate in the OC group, with the assumption that 30% of those invited would adhere to the invitation. This assumption is based on data from the SCORE3 trial, where adherence to colonoscopy in the Florence district was 27.9% [22]. In the CTC group we invited 5,000 subjects with an expected participation of 35%. Thus it will be possible to detect as statistically significant a difference of more than 3.1% with a two-tailed test and of more than 2.6% with a one-tailed test, setting the value of the alpha error equal to 5% (power = 0.80).

#### Comparison of CTC with reduced preparation versus CTC with standard preparation

A total of 2,500 subjects were invited to participate in the CTC subgroup with reduced preparation, and 2,500 subjects were invited to participate in the CTC subgroup with standard preparation. In this way, it will be possible to detect, as statistically significant, a difference of more than 2.6% with two-tailed test and of more than 2.2% with a one-tailed test, by setting the value of the alpha error equal to 5% (power = 0.80).

#### Estimation of the recall rate to OC in the CTC arm

With a sample size of 5,000 subjects we will be able to estimate the recall rate to OC (assuming a true positivity rate equal to 15%) with an uncertainty of +/−2.5%. (alpha = 0.05; power = 0.80).

#### Comparison of detection rate for colonic lesions between a single round of CTC and three rounds of FOBT

A detection rate for advanced adenomas or carcinomas equal to 5% is expected for CTC. In the group of 8,000 subjects invited to undergo FOBT, an adherence to invitation of about 50% was assumed. If the cumulative detection rate for advanced adenomas and carcinomas of three FOBT rounds will be less than 4%, a statistically significant difference (alpha = 0.05, power = 0.80, one tail) with the detection rate of a single round of CTC will be revealed.

## Discussion

FOBT and FS are currently recommended for population-based screening programs in Italy, and are employed in several regions [16]. Moreover, the European Guidelines on quality assurance for CRC screening has recently recommended FIT as the first choice test in place of the guaiac test [41].

CTC has been demonstrated to be an accurate technique that permits a minimally invasive structural evaluation of the whole colon [23-29]. In our study we will basically compare diagnostic yield of CTC versus three rounds of biannual FIT. In fact, efficacy of FOBT in reducing mortality from CRC relies on the cumulative sensitivity that the test offers round by round. Thus, a correct assessment of diagnostic yield of the two screening strategies has to be carried out comparing a single CTC examination with a number of FOBT rounds approximate to the suggested rescreening interval of CTC, namely five years [11,12].

So far, data about adherence to CTC invitation in comparison with FIT are not available in the setting of a population-based screening program. Many other aspects concerning a potential role of CTC as first level screening test have not been evaluated yet. These include the impact of referral rate to OC in CTC positive subjects and the estimate of OC workload and costs of CTC screening as compared to both OC and FOBT-based screening.

Acceptability represents a critical determinant of the impact of a screening program. High participation of invited people can be obtained if the offered screening test is well accepted. The acceptability of CTC can be enhanced if the examination is performed without an extensive bowel preparation as the one required for OC [42,43]. Up-to-date large trials evaluating accuracy of CTC in asymptomatic average risk subjects were conducted either using full laxative preparation with fecal tagging or employing reduced bowel preparation [24,25,44]. Also the Dutch study used a limited bowel preparation [36]. Thus, it is of interest to evaluate how different bowel preparations influence attendance to CTC screening.

In our study we utilize a teleradiological model of CTC reading, which consists of performing the examination in the hospitals near the citizen’s house and sending datasets via the RIS/PACS metropolitan area network to a centralized reporting unit. Reading centralization is expected to improve the quality of a radiologist’s reading performance. It will be also of interest to evaluate how the availability of the test near the subject’s house could enhance attendance.

In our study CTC will be interpreted using a CAD system as the first reader. This reading modality could reduce reading time and have a positive impact on costs of screening with CTC.

We will also compare CTC and FIT screening with OC as a first-level test. OC is widely accepted as the diagnostic gold standard for the detection of CRC and as such represents the mandatory second-level examination after positive FOBT or CTC screening tests. However OC has a low acceptance among individuals at average risk of the disease, and this critical aspect hinders its use as a primary screening test [22].

Our study investigating CTC as a primary screening test in a population-based program of CRC prevention will provide information about participation/acceptability, diagnostic yield and costs of this test in comparison with the recommended test (FOBT) and with OC.

## Trial status

The trial started recruitment in January 2013.

## Abbreviations

CAD: Computer-aided diagnosis; C-RADS: CT Colonography reporting and data system; CRC: Colorectal cancer; CTC: Computed tomography colonography; DICOM: Digital imaging and communications in medicine; FIT: Fecal immunochemical test; FOBT: Fecal occult blood test; FS: Flexible sigmoidoscopy; ISPO: Cancer research and prevention institute; OC: Optical colonoscopy; RCT: Randomized clinical trial.

## Competing interests

The authors declare that they have no competing interests.

## Authors’ contributions

LS, GG, MZ and MM are responsible for the drafting of the manuscript. All authors are responsible for the study design and revision of the manuscript. All authors have read and approved the manuscript.

## References

[B1] Ferlay J Parkin DM Steliarova-Foucher E Estimates of the cancer incidence and mortality in Europe in 2008 EJC 2010 46 765 781 10.1016/j.ejca.2009.12.014 20116997

[B2] Crocetti E Buzzoni C AIRTUM Working Group New incidence and mortality data. 2003–2005 Epidemiol Prev 2009 Suppl 2 e1 e3 e5-e26 19773603

[B3] Gondos A Bray F Hakulinen T Brenner H Trends in cancer survival in 11 European populations from 1990 to 2009: a model-based analysis Ann Oncol 2009 20 564 573 19066327 10.1093/annonc/mdn639

[B4] Winawer SJ Zauber AG Ho MN O'Brien MJ Gottlieb LS Sternberg SS Waye JD Schapiro M Bond JH Panish JF Ackroyd F Shike M Kurtz RC Hornsby-Lewis L Gerdes H Stewart ET The National Polyp Study Workgroup Prevention of colorectal cancer by colonoscopic polypectomy. The national polyp study workgroup N Engl J Med 1993 329 1977 1981 10.1056/NEJM199312303292701 8247072

[B5] Mandel JS Bond JH Church TR Snover DC Bradley GM Schuman LM Ederer F Reducing mortality from colorectal cancer by screening for fecal occult blood. Minnesota colon cancer control study N Engl J Med 1993 328 1365 1371 10.1056/NEJM199305133281901 8474513

[B6] Hardcastle JD Chamberlain JO Robinson MH Moss SM Amar SS Balfour TW James PD Mangham CM Randomised controlled trial of faecal-occult-blood screening for colorectal cancer Lancet 1996 348 1472 1477 10.1016/S0140-6736(96)03386-7 8942775

[B7] Kronborg O Fenger C Olsen J Jorgensen OD Sondergaard O Randomised study of screening for colorectal cancer with faecal-occult-blood test Lancet 1996 348 1467 1471 10.1016/S0140-6736(96)03430-7 8942774

[B8] Atkin WS Edwards R Kralj-Hans I Wooldrage K Hart AR Northover JM Parkin DM Wardle J Duffy SW Cuzick J UK Flexible Sigmoidoscopy Trial Investigators Once-only flexible sigmoidoscopy screening in prevention of colorectal cancer: a multicentre randomised controlled trial Lancet 2010 375 1624 1633 10.1016/S0140-6736(10)60551-X 20430429

[B9] Segnan N Armaroli P Bonelli L Risio M Sciallero S Zappa M Andreoni B Arrigoni A Bisanti L Casella C Crosta C Falcini F Ferrero F Giacomin A Giuliani O Santarelli A Visioli CB Zanetti R Atkin WS Senore C SCORE Working Group Once-only sigmoidoscopy in colorectal cancer screening: follow-up findings of the Italian randomized controlled trial – SCORE J Natl Cancer Inst 2011 103 1310 1322 10.1093/jnci/djr284 21852264

[B10] Schoen RE Pinsky PF Weissfeld JL Yokochi LA Church T Laiyemo AO Bresalier R Andriole GL Buys SS Crawford ED Fouad MN Isaacs C Johnson CC Reding DJ O'Brien B Carrick DM Wright P Riley TL Purdue MP Izmirlian G Kramer BS Miller AB Gohagan JK Prorok PC Berg CD the PLCO Project Team Colorectal-cancer incidence and mortality with screening flexible sigmoidoscopy N Engl J Med 2012 366 2345 2357 10.1056/NEJMoa1114635 22612596 PMC3641846

[B11] Levin B Lieberman DA McFarland B Andrews KS Brooks D Bond J Dash C Giardiello FM Glick S Johnson D Johnson CD Levin TR Pickhardt PJ Rex DK Smith RA Thorson A Winawer SJ American Cancer Society Colorectal Cancer Advisory Group Screening and surveillance for the early detection of colorectal cancer and adenomatous polyps, 2008: a joint guideline from the American Cancer Society, the US Multi-Society Task Force on Colorectal Cancer, and the American College of Radiology Gastroenterology 2008 134 1570 1595 10.1053/j.gastro.2008.02.002 18384785

[B12] Rex DK Johnson DA Anderson JC Schoenfeld PS Burke CA Inadomi JN American college of gastroenterology guidelines for colorectal cancer screening 2008 Am J Gastroenterol 2009 104 739 750 10.1038/ajg.2009.104 19240699

[B13] Castiglione G Zappa M Grazzini G Rubeca T Turco P Sani C Ciatto S Screening for colorectal cancer by faecal occult blood test: comparison of immunochemical tests J Med Screen 2000 7 35 37 10.1136/jms.7.1.35 10807145

[B14] Morikawa T Kato J Yamaji Y Wada R Mitsushima T Shiratori Y A comparison of the immunochemical fecal occult blood test and total colonoscopy in the asymptomatic population Gastroenterology 2005 129 422 428 16083699 10.1016/j.gastro.2005.05.056

[B15] van Rossum LG van Rijn AF Laheij RJ van Oijen MG Fockens P van Krieken HH Verbeek AL Jansen JB Dekker E Random comparison of guaiac and immunochemical fecal occult blood tests for colorectal cancer in a screening population Gastroenterology 2008 135 82 90 10.1053/j.gastro.2008.03.040 18482589

[B16] Zorzi M Falcini F Fedato C Grazzini G de’ Bianchi PS Senore C Vettorazzi M Visioli C Zappa M Screening for colorectal cancer in Italy: 2006 survey Epidemiol Prev 2008 32 55 68 18770995

[B17] Grazzini G Castiglione G Ciabattoni C Franceschini F Giorgi D Gozzi S Mantellini P Lopane P Perco M Rubeca T Salvadori P Visioli CB Zappa M Colorectal cancer screening programme by faecal occult blood test in Tuscany: first round results Eur J Cancer Prev 2004 13 19 26 10.1097/00008469-200402000-00004 15075784

[B18] Istituto per lo Studio e la Prevenzione Oncologica (ISPO) http://www.cspo.it/

[B19] Singh H Turner D Xue L Targownik LE Bernstein CN Risk of developing colorectal cancer following a negative colonoscopy examination: evidence for a 10-year interval between colonoscopies JAMA 2006 295 2366 2373 10.1001/jama.295.20.2366 16720822

[B20] Brenner H Chang-Claude J Seiler CM Hoffmeister M Long-term risk of colorectal cancer after negative colonoscopy J Clin Onco 2011 29 3761 3767 10.1200/JCO.2011.35.9307 21876077

[B21] Nelson DB McQuaid KR Bond JH Lieberman DA Weiss DG Johnston TK Procedural success and complications of large-scale screening colonoscopy Gastrointest Endosc 2002 55 307 314 10.1067/mge.2002.121883 11868001

[B22] Segnan N Senore C Andreoni B Azzoni A Bisanti L Cardelli A Castiglione G Crosta C Ederle A Fantin A Ferrari A Fracchia M Ferrero F Gasperoni S Recchia S Risio M Rubeca T Saracco G Zappa M SCORE3 Working Group-Italy Comparing attendance and detection rate of colonoscopy with sigmoidoscopy and FIT for colorectal cancer screening Gastroenterology 2007 132 2304 2312 10.1053/j.gastro.2007.03.030 17570205

[B23] Pickhardt PJ Hassan C Halligan S Marmo R Colorectal cancer: CT colonography and colonoscopy for detection–systematic review and meta-analysis Radiology 2011 259 393 405 10.1148/radiol.11101887 21415247 PMC3079122

[B24] Pickhardt PJ Choi JR Hwang I Butler JA Puckett ML Hildebrandt HA Wong RK Nugent PA Mysliwiec PA Schindler WR Computed tomographic virtual colonoscopy to screen for colorectal neoplasia in asymptomatic adults N Engl J Med 2003 349 2191 2200 10.1056/NEJMoa031618 14657426

[B25] Johnson CD Chen MH Toledano AY Heiken JP Dachman A Kuo MD Menias CO Siewert B Cheema JI Obregon RG Fidler JL Zimmerman P Horton KM Coakley K Iyer RB Hara AK Jr Halvorsen RA Casola G Yee J Herman BA Burgart LJ Limburg PJ Accuracy of CT colonography for detection of large adenomas and cancers N Engl J Med 2008 359 1207 1217 10.1056/NEJMoa0800996 18799557 PMC2654614

[B26] Kim DH Pickhardt PJ Taylor AJ Leung WK Winter TC Hinshaw JL Gopal DV Reichelderfer M Hsu RH Pfau PR CT colonography versus colonoscopy for the detection of advanced neoplasia N Engl J Med 2007 357 1403 1412 10.1056/NEJMoa070543 17914041

[B27] Burling D Halligan S Slater A Noakes MJ Taylor SA Potentially serious adverse events at CT colonography in symptomatic patients: national survey of the United Kingdom Radiology 2006 239 464 471 10.1148/radiol.2392051101 16569789

[B28] Sosna J Blachar A Amitai M Barmeir E Peled N Goldberg SN Bar-Ziv J Colonic perforation at CT colonography: assessment of risk in a multicenter large cohort Radiology 2006 239 457 463 10.1148/radiol.2392050287 16543590

[B29] Pickhardt PJ Incidence of colonic perforation at CT colonography: review of existing data and implications for screening of asymptomatic adults Radiology 2006 239 313 316 10.1148/radiol.2392052002 16641348

[B30] Jensch S Bipat S Peringa J de Vries AH Heutinck A Dekker E Baak LC Montauban van Swijndregt AD Stoker J CT colonography with limited bowel preparation: prospective assessment of patient experience and preference in comparison to optical colonoscopy with cathartic bowel preparation Eur Radiol 2010 20 146 156 10.1007/s00330-009-1517-0 19626326 PMC2803752

[B31] Liedenboum MH Thenema HW Stoker J Radiation dose in CT colonography-trends in time and differences between daily practice and screening protocols Eur Radiol 2008 18 2222 2230 10.1007/s00330-008-0994-x 18491095

[B32] Pickhardt PJ Hanson ME Vanness DJ Lo JY Kim DH Taylor AJ Winter TC Hinshaw JL Unsuspected extracolonic findings at screening CT colonography: clinical and economic impact Radiology 2008 249 151 159 10.1148/radiol.2491072148 18796673

[B33] Summers Jerebko AK Franaszek M Malley JD Johnson CD Colonic Polyp: complementary role of computer aided detection in CT colonography Radiology 2002 255 391 399 12409571 10.1148/radiol.2252011619

[B34] Mani A Napel S Paik DS Jr Jeffrey RB Yee J Olcott EW Prokesch R Davila M Schraedley-Desmond P Beaulieu CF Computed tomography colonography: feasibility of computer-aided polyp detection in a first-reader paradigm J Comput Assist Tomogr 2004 28 318 332 10.1097/00004728-200405000-00003 15100534

[B35] Edwards JT Mendelson RM Fritschi L Foster NM Wood C Murray D Forbes GM Colorectal Neoplasia Screening with CTC colonography in average-risk asymptomatic subjects: community based study Radiology 2004 230 459 464 10.1148/radiol.2302021422 14688402

[B36] Stoop EM de Haan MC de Wijkerslooth TR Bossuyt PM van Ballegooijen M Nio CY van de Vijver MJ Biermann K Thomeer M van Leerdam ME Fockens P Stoker J Kuipers EJ Dekker E Participation and yield of colonoscopy versus non-cathartic CT colonography in population-based screening for colorectal cancer: a randomised controlled trial Lancet Oncol 2012 13 55 64 10.1016/S1470-2045(11)70283-2 22088831

[B37] Zalis ME Barish MA Choi JR Dachman AH Fenlon HM Ferrucci JT Glick SN Laghi A Macari M McFarland EG Morrin MM Pickhardt PJ Soto J Yee J Working Group on Virtual Colonoscopy CT colonography reporting and data system: a consensus proposal Radiology 2005 236 3 9 10.1148/radiol.2361041926 15987959

[B38] Schlemper RJ Riddell RH Kato Y Borchard F Cooper HS Dawsey SM Dixon MF Fenoglio-Preiser CM Fléjou JF Geboes K Hattori T Hirota T Itabashi M Iwafuchi M Iwashita A Kim YI Kirchner T Klimpfinger M Koike M Lauwers GY Lewin KJ Oberhuber G Offner F Price AB Rubio CA Shimizu M Shimoda T Sipponen P Solcia E Stolte M Watanabe H Yamabe H The Vienna classification of gastrointestinal epithelial neoplasia Gut 2000 47 251 255 10.1136/gut.47.2.251 10896917 PMC1728018

[B39] Trouet C New European guidelines for the use of stored human biological materials in biomedical research J Med Ethics 2004 30 99 103 10.1136/jme.2003.003467 14872085 PMC1757121

[B40] Senore C Ederle A Fantin A Andreoni B Bisanti L Grazzini G Zappa M Ferrero F Marutti A Giuliani O Armaroli P Segnan N Acceptability and side-effects of colonoscopy and sigmoidoscopy in a screening setting J Med Screen 2011 18 128 134 10.1258/jms.2011.010135 22045821

[B41] Halloran S Launoy G Zappa M Segnan N, Patnick J, von Karsa L Faecal occult blood testing European Guidelines for Quality Assurance in Colorectal Cancer Screening and Diagnosis 2010 1 Luxembourg: Publication Office of the European Union 103 144

[B42] Taylor SA Slater A Burling DN Tam E Greenhalgh R Gartner L Scarth J Pearce R Bassett P Halligan S CT colonography: optimisation, diagnostic performance and patient acceptability of reduced-laxative regimens using barium-based faecal tagging Eur Radiol 2008 18 32 42 10.1007/s00330-007-0631-0 17404739 PMC2220024

[B43] Campanella D Morra L Delsanto S Tartaglia V Asnaghi R Bert A Neri E Regge D Comparison of three different iodine-based bowel regimens for CT colonography Eur Radiol 2010 20 348 358 10.1007/s00330-009-1553-9 19711082

[B44] Zalis ME Blake MA Cai W Hahn PF Halpern EF Kazam IG Keroack M Magee C Näppi JJ Perez-Johnston R Saltzman JR Vij A Yee J Yoshida H Diagnostic accuracy of laxative-free computed tomographic colonography for detection of adenomatous polyps in asymptomatic adults: a prospective evaluation Ann Intern Med 2011 156 692 702 22586008 10.7326/0003-4819-156-10-201205150-00005

